# Food environment in Burkina Faso: priority actions recommended to the government using Food-EPI tool

**DOI:** 10.3389/fnut.2024.1420323

**Published:** 2024-07-18

**Authors:** Viviane Aurelie Tapsoba, Ella W. R. Compaore, Augustin Nawidimbasba Zeba, Jerome Winbetourefa Some, Julien Soliba Manga, Adama Diouf, Jean-Claude Moubarac, Stefanie Vandevijvere, Mamoudou Hama Dicko

**Affiliations:** ^1^Laboratory of Biochemistry, Biotechnology, Food Technology and Nutrition (LABIOTAN), Department of Biochemistry and Microbiology, Joseph KI-ZERBO University, Ouagadougou, Burkina Faso; ^2^Research Institute for Health Sciences (IRSS), Bobo Dioulasso, Burkina Faso; ^3^Research Institute for Health Sciences (IRSS), Ouagadougou, Burkina Faso; ^4^Department of Nutrition, Montreal University, Québec, QC, Canada; ^5^Laboratory for Research into Human Nutrition and Food (LARNAH), Department of Animal Biology, Faculty of Science and Technology, Cheikh Anta Diop University (UCAD), Dakar, Senegal; ^6^Nutrition and Health, Sciensano, Brussels, Belgium

**Keywords:** food environment, Food-EPI, food system, government actions, public policies, Burkina Faso

## Abstract

**Introduction:**

The food environment is an important factor in the efforts of countries worldwide to achieve a transition to sustainable food systems. The objective of this study is to formulate and prioritize actions to be addressed to the government of Burkina Faso for the creation of a healthy food environment, which will contribute to reducing malnutrition in all its forms and non-communicable diseases.

**Methods:**

National experts were brought together to identify and prioritize actions to fill the gaps identified through a multi-step assessment process following the methodology of the Healthy Food and Environment Policy Index (Food-EPI).

**Results:**

Up to 20 priority policy actions were recommended to the Burkina Faso government. Actions in the policy component focused mainly on regulation of food promotion and marketing, particularly to children, and others in the infrastructure support component focused largely on political leadership, i.e., strong and visible political support from the government to improve the food environment, population nutrition, diet-related non-communicable diseases and their inequalities.

**Conclusion:**

The priority actions to be recommended to the government will strengthen advocacy for government decisions to create a healthier food environment in the country.

## 1 Introduction

Over the past decade, highly processed, energy-intensive, micronutrient-poor foods have become more promoted. They are accessible and inexpensive compared to less accessible fresh, minimally processed or unprocessed foods (1).

Nutritional transition is defined as changes in diets at the population level, corresponding to globalization and changes in a country's overall development, food environments and food systems. Risk factors such as poor-quality diet have always been recognized as important for overweight and obesity (2, 3). These factors are incorporated into everyday life, partly as a result of various exposures, particularly the food environment (4).

The food environment, which is characterized by the physical, economic, political, and socio-cultural conditions that influence dietary decisions and marketing strategies, is a key factor in the spread of unhealthy eating, and a major risk factor for all forms of malnutrition (5–7). Burkina Faso's actual food environments are similar to those of the global food environment in that they do not support the consumption of healthy, sustainable diets and are therefore subject to increasingly high prevalence of all forms of malnutrition and non-communicable diseases.

Based on the 2021 Burkina Faso national nutrition survey, the prevalence of global acute malnutrition has fallen from 11.3% to 9.7% (including 0.8% in the severe form), chronic malnutrition from 35.1% to 21.6% and underweight from 26% to 17.5% (8). Overweight and obesity affected 0.9% and 0.2% of children respectively (8). The national prevalence of overweight and obesity among reproductive-age women in 2021 was 12.3% and 6.4% respectively (8). Among teenage girls aged 10 to 19, 0.6% were overweight and 4.7% obese. % (8). According to the report of the second national survey on the prevalence of the main common risk factors for non-communicable diseases in Burkina Faso, compared with 2013, the prevalence of obesity and overweight among men and women increased in 2021. It should be noted that after 2021, there are no national estimates because not all regions could be surveyed due to the security situation. In addition to these different forms of malnutrition, micronutrient deficiencies and the development of non-communicable diseases are still a major concern. The prevalence of anemia in children under five was 41% in 2020 (9). The prevalence of hypertension has risen from 17.6% to 18.2% and that of diabetes from 4.9% to 7.6% between 2013 and 2021 (10). However, the data from these results show that undernutrition and overnutrition coexist in the country, requiring the implementation of appropriate policies and programs to deal with them.

In a context where national governments are the main actors with the greatest capacity to change food environments and people's diets (11, 12), sustainable nutritional solutions therefore require appropriate policies and systems (13). Implementing effective policies can improve food environments, which in turn improve the nutritional status of the population and prevent overweight, obesity, and diet-related non-communicable diseases.

In Burkina Faso, the government's commitment to improving the nutritional status of the population through various measures is an expression of its interest in creating a healthy food environment. Indeed, in terms of food and nutrition policy, the country has recently set up a multi-sectoral and multi-disciplinary nutrition platform, which represents an opportunity to facilitate the evaluation of public policies and the promotion of political actions aimed at improving the nutrition of populations in Burkina Faso (14).

This study on food environments is a first in Burkina Faso, and has seen several innovations in the implementation of the Food EPI tool. A major innovation in this research is the addition of 12 new indicators taking into account the double nutritional burden, namely breastfeeding and complementary feeding, regulations on the marketing of breast-milk substitutes (MMS), national policies to combat overweight, NCDs; undernourishment, health systems (growth monitoring); hygiene, water and sanitation (WASH); and sanitary safety (microbial and chemical contamination) (15). There is also an innovation in the methodology concerning the prioritization criteria in the process. In fact, 2 new criteria have been added, making it possible to take into account the gender and sustainability of the political actions to be prioritized for the creation of food environments.

The aim of this study was to formulate and prioritize policy actions relating to a healthy food environment in Burkina Faso, in order to reduce overweight, obesity, or non-communicable diseases. The first part of this paper describes the methodology applied, followed by the results presentation and discussion.

## 2 Methods

### 2.1 The socio-economic and demographic characteristics of Burkina Faso

Burkina Faso is a landlocked country located in the heart of West Africa. The country is exposed to a number of obstacles that hamper its economic development. The main factors limiting its development range from the precarity of rainfall to the remoteness of the sea, not to mention the low level of technology use in its agriculture sector that employs the majority of the population (30% of GDP, 80% of jobs).

According to the latest Census conducted in 2019, the literacy rate for people aged 15 and over was estimated at 29.7% nationwide, and 55.6% in urban areas compared to 18.5% in rural areas.

Based on the Human Development Index 2020 report, the country ranks 182 out of 189 countries, and according to the World Bank, 40.1% of the population lives below the poverty line (16). In terms of urbanization, Burkina Faso, like many developing countries, has experienced rapidly growing urbanization, from 6.4% in 1975 to 26.3% in 2019, although these urbanization rates remain among the lowest in the sub-region.

### 2.2 Study setting

This study used the healthy food environment policy index (Food-EPI) module developed in 2012 by the international network for food and obesity/non communicable diseases research, monitoring and action support (INFORMAS). This module comprises a tool and a process that have been designed to make inquiry on what progress the government has made in good practice to improve food environments and implement policies and actions to prevent obesity and non-communicable diseases (17, 18).

The implementation of the Food-EPI process may enable the identification and prioritization of government actions for the creation of food environments in Burkina Faso (17, 19). To accomplish this, the first step was to identify public policies and government actions using the Food-EPI tool, followed by a second step to assess the level of implementation of food environment and infrastructure support policies. This was followed by the implementation of the third step, in which key government recommendations were identified and prioritized by a panel of multidisciplinary operational experts in the field of nutrition.

### 2.3 Data sources and collection

The tool and process established by the Food-EPI module was the theoretical framework that guided data collection and analysis for this study in Burkina Faso.

#### 2.3.1 Food-EPI tool

The Food-EPI tool comprises seven policy domains representing key aspects of food environments: food composition, food labeling, food promotion, food pricing, food supply, food retailing and food trade and investment, and six infrastructure support domains: leadership, governance, finance and resources, monitoring, and evaluation, interaction platforms and health in all policies. The Food-EPI tool was adapted to the Burkinabe context by the research team in charge of implementing the tool in the country, contextualizing the original Food-EPI protocol (17).

Thus, the original 47-indicators tool has undergone some modifications. “3” indicators have been deleted: “2” indicators associated with “food retailing” and “1” indicator associated with “health in all policies.” These three indicators have been removed because they were similar to existing indicators.

The Food-EPI tool has been implemented in a number of sub-Saharan countries, including Ghana, Kenya and Senegal (20–22). A key recommendation from stakeholders involved in the Food-EPI process in these countries was to make Food-EPI indicators sensitive to the creation of healthy food environments to combat undernutrition (e.g., micronutrient deficiencies, stunting, and acute malnutrition), as these are a major public health problem in sub-Saharan Africa.

These new indicators are also in line with WHO recommendations for double-duty actions to combat all forms of malnutrition (15). Following these recommendations, in 2020 the INFORMAS team and researchers involved in research on food environments began the process of developing relevant indicators of undernutrition, to be included in the Food-EPI tool.

**Figure 1:**
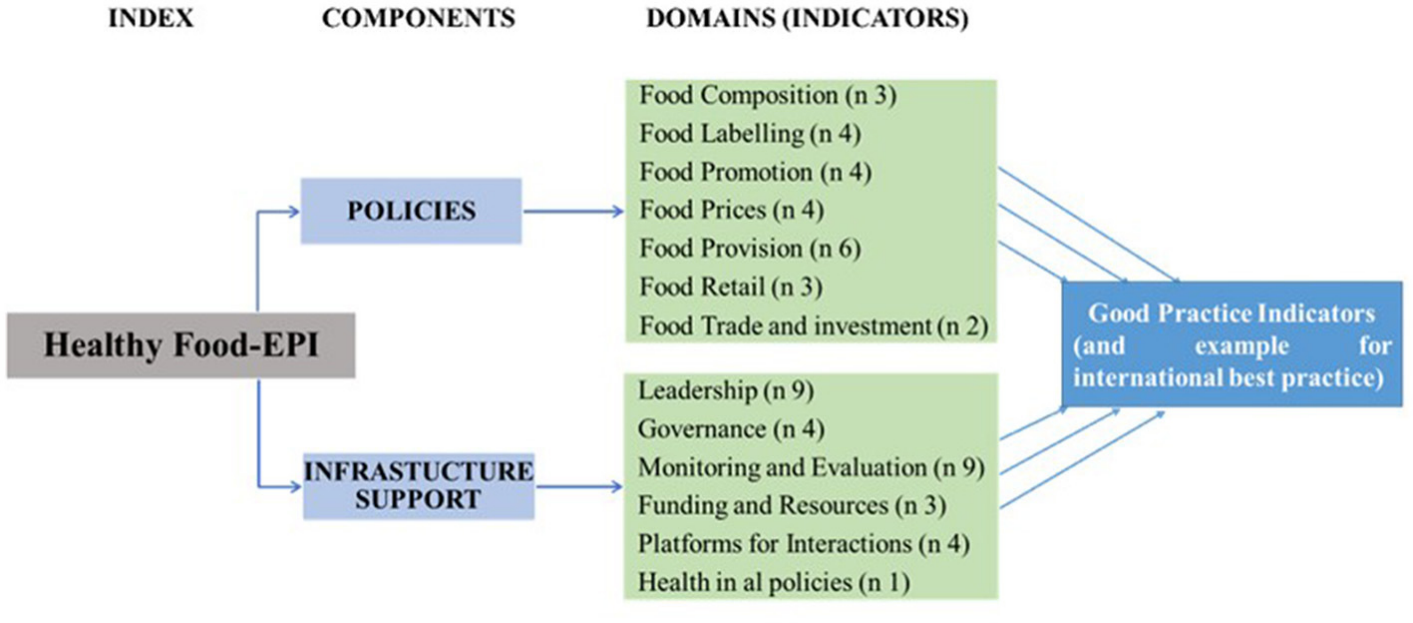
Components and domains of the Food-EPI adapted to the Burkina Faso context (18).

Then, 12 new double burden of malnutrition indicators have been added, relating to breastfeeding and complementary feeding, regulations on the marketing of breast-milk substitutes, national policies to combat overweight, NCDs and undernutrition, health systems (growth monitoring), water, sanitation, and hygiene (WASH) indicators, food retailers, and traders (hygiene and sanitation), and health safety (microbial and chemical contamination) (23). The final Food-EPI tool in Burkina Faso comprised 56 indicators grouped into 13 policy and infrastructure support domains (Figure 1) (see details in Appendix 1).

#### 2.3.2 Food-EPI implementation process

**Figure 2:**
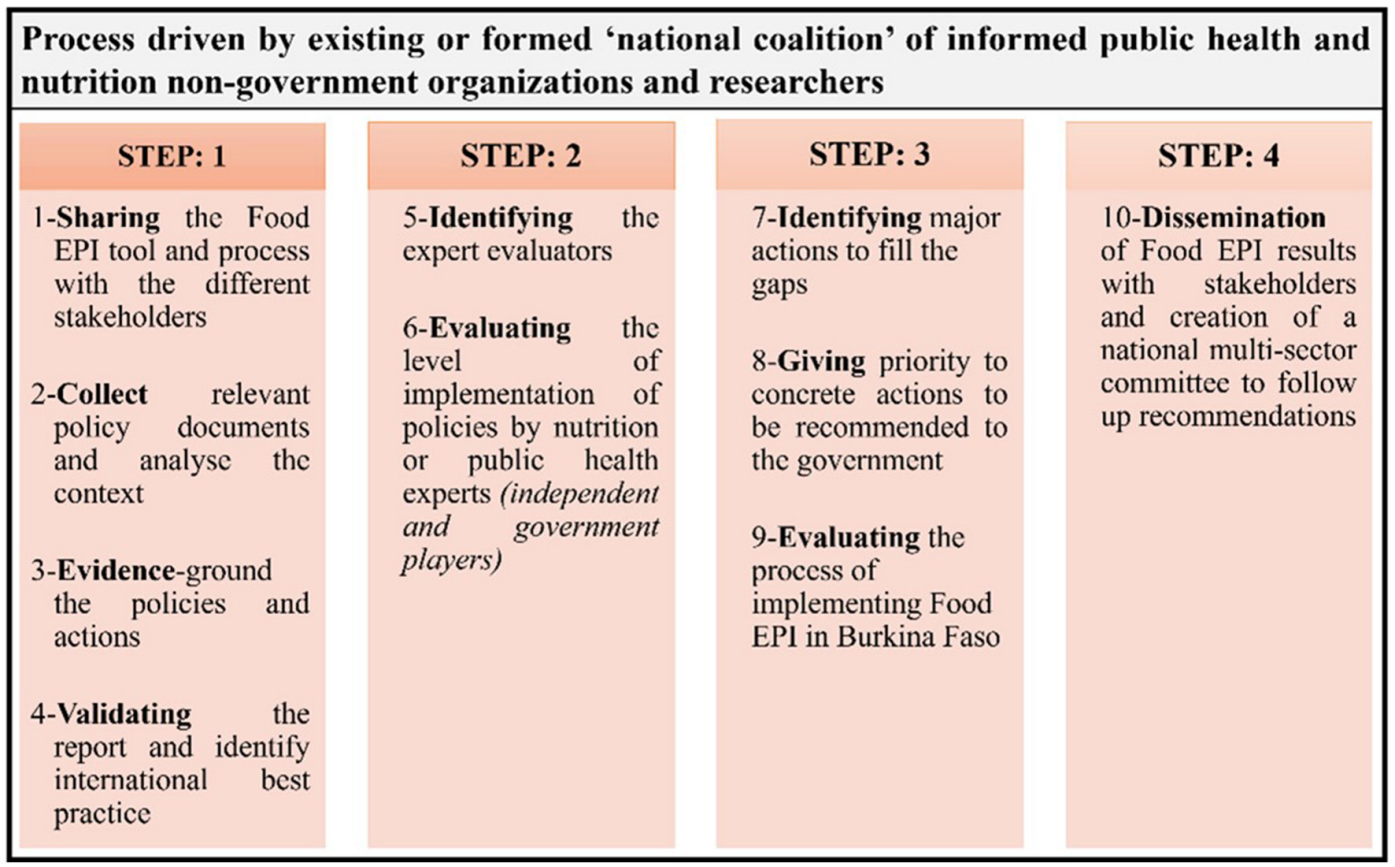
Process for assessing the level of implementation of government policy on food environments in Burkina Faso, 2022.

The process was divided into six steps, which have been implemented in previous studies (24, 25), with the exception of the last two step concerning the formulation and prioritization of actions, which was the subject of this study (Figure 2). Afterwards, evidence on the implementation of public policies and government actions was collected, summarized in an evidence document and verified by government staff in Burkina Faso.

Information collected on policies and governmental actions during the implementation of the Food-Epi process and used as secondary data in this study included: (1) Policy documents: policy guidelines on nutrition and health or legislations (law or decree) in the field of nutrition; (2) Strategic documents: documents outlining political or strategic axes or operationalizing policy guidelines; and (3) Operational documents comprising: (a) reports on the activities of various sectors that are sensitive or specific to nutrition, (b) reports on national nutrition or health surveys, and (c) nutrition programs and food-related guides.

On the basis of this evidence document, the level of implementation of food environmental policies was assessed against international examples of good practice. Following this final assessment, actions recommended to the government were formulated by experts and prioritized according to their importance, feasibility, effect on double burden, gender and sustainability.

#### 2.3.3 Panel of expert evaluators

In line with Food-EPI's methodology, the process of identifying and prioritizing actions was organized throughout a physical presence workshop.

In order to formulate and prioritize the actions to be recommended to governments, the invited participants to this workshop were selected according to their field of expertise, while ensuring that they had already participated in the majority of steps in the overall Food-EPI process. These expert evaluators were from universities, government organizations, civil society, and Non-Governmental Organizations involved in public health and/or nutrition.

### 2.4 Identifying and prioritizing actions

The process of identifying and prioritizing actions took the form of three-step workshops: first, the identification, then the prioritization of actions, and finally the priority actions to be recommended to the government.

#### 2.4.1 Action identification process

This step was a 1-day workshop, during which the results of the assessment of the level of implementation of policy was presented, in order to facilitate the identification and formulation of actions.

The main outcome was the selection of actions to fill identified gaps in policy implementation, to reinforce already implemented policy actions, or to select actions that could address the problems of undernourishment by improving food environments. This identification of actions was carried out by two mixed working groups. Each group identified actions by component, leaving it to each group to decide whether an indicator should include several actions or none at all.

At the end of this step, the identified actions by each group were presented and discussed, and a common list of concrete actions to be prioritized was validated by all the experts.

#### 2.4.2 Action prioritization process

This step followed that of formulating actions and consisted in prioritizing individually the actions by each expert, using a Likert scale from 1 to 5. This prioritization of validated actions was carried out according to five criteria (Table 1): (i) importance of the action, (ii) capacity to carry out the action, (iii) potential effect of the action on double burden, (iv) potential effect of the action on gender and (v) potential effect of the action on sustainability.

**Table 1:** Criteria for prioritizing actions to be recommended to the government.

**Criterion 1 (C1): importance**	**1. Need:** Size of implementation gap **2. Impact:** the effectiveness of the action in improving food environments **3. Effects on equity and other positive or negative effects of the action:** ✓ Equity: Progressive/regressive effects on reducing health inequalities linked to food and diet. ✓ Other positive effects (example): Protection of children's and consumers' rights ✓ Negative effects (example): regressive effects on household income or violation of personal freedoms
**Criterion 2 (C2): ability to perform**	**1. Feasibility:** How easy or difficult will it be to implement the action? **2. Acceptability:** The level of support from key stakeholders (government, public, public health and industry). **3. Affordability:** The cost of implementing the action
**Criterion 3 (C3): potential impact of the action on the double burden of malnutrition**	**1. Beneficial effect:** Implementation of the action has a beneficial effect on the double burden of malnutrition **2. Aggravating effect:** Implementation of the action increases the risk of other forms of malnutrition or NCDs **3. Neutral effect:** Implementation of the action has no effect on increasing the risk of other forms of malnutrition or NCDs.
**Criterion 4 (C4): potential gender impact of the action**	**1. Beneficial effect:** Implementation of the action has a beneficial effect on gender. **2. Aggravating effect:** Implementation of the action increases the risk of other forms of gender inequality **3. Neutral effect:** The implementation of the action has no effect on gender inequalities.
**Criterion 5 (C5): potential impact of the action on sustainability**	**1. Beneficial effect:** Implementation of the action has a beneficial effect on sustainability. **2. Aggravating effect:** Implementation of the action increases the risk of adverse effects on sustainability **3. Neutral effect:** Implementation of the action has no effect on sustainability.

An interesting outcome was found in the implementation of Food-EPI in Burkina Faso. Indeed, in addition to the two initial criteria (C1 and C2) of the Food-EPI module and one introduced during the implementation of Food-EPI in Senegal (C3) (22), this study introduced two new prioritization criteria (C4 and C5). These criteria took into account the potential effect of the action on gender and sustainability, which are two public health issues strongly influenced by socio-economic, demographic and climate change.

#### 2.4.3 Actions to be recommended to the government

After prioritizing the actions on the basis on the five criteria, a final step determined the main actions to be recommended to the government.

This was the step of disseminating the results. This final stage in the implementation process of the Food-EPI research project will be carried out in the form of a workshop, and will provide an opportunity to synthesize the data from the results of the identification and prioritization step.

At this step, the actions to be recommended to the government will be gone through, and in a common consensus with the parties involved they will be reformulated or combined in order to broadly take into account all the needs. Then 20 priority actions based on all the criteria will be validated, among which 10 from policy component actions and 10 from infrastructure support actions.

### 2.5 Data analysis

Descriptive statistics performed using Microsoft Excel 2021. To prioritize proposed actions, individual scores were assigned to the importance, feasibility, and potential effect of the action on the double burden of malnutrition, gender and sustainability.

The average scores for each criteria was then merged for each action to determine a single criteria corresponding to “priority actions.” In this way, actions were classified from the highest to lowest priority.

### 2.6 Ethical considerations

This study was reviewed and approved by the Burkina Faso health research ethics committee under deliberation no. 2021-04-112. In order to facilitate data collection, an administrative letter was sent by the Ministry of Health through the Technical Secretariat in charge of multisectoral nutrition to all the ministries concerned. All ministries and stakeholders involved in nutrition were informed of the purpose of the study. The ethical consideration was taken into account by respecting the anonymity of the actors during data analysis.

## 3 Results

### 3.1 Expert evaluators

Among 56 invited participants, 40 expert evaluators were physically present, giving a participation rate of 71.4%. Among evaluators, 17 were independent stakeholders and 23 were staff from government.

### 3.2 Policy actions and support for identified and prioritized infrastructures

After calculating the average score for each of the actions prioritized by all the experts (see details in Appendix 2), they were ranked in descending order according to the average score obtained by adding together the five prioritization criteria. Each action was ranked according to all criteria, from the most important action, with a high capacity for implementation, a high potential beneficial effect on the double burden, gender and sustainability, to the least important action, with a low capacity for implementation, a low potential effect on the double burden, gender and sustainability.

This workshop was held from June 20 to 21, 2022 and the expert evaluators identified 123 priority actions to improve the food environment in Burkina Faso, including 70 actions under the “policy” component and 53 actions under the “infrastructure support” component (see details in Appendix 2). These identified policy actions concerned all macro and micro level.

### 3.3 Actions to be recommended to the government

Based on the five criteria used in this study, 10 priority actions from each component to be recommended to the government were selected by mutual agreement, taking into account the experts' perception of the importance, feasibility, potential effect on the double burden of malnutrition, gender and sustainability of these actions in Burkina Faso (Tables 2, 3).

**Table 2:** Priority policy actions according to all criteria to be recommended to the government of Burkina Faso, 2023.

**Order number**	**Indicators concerned**	**Priority actions recommended to the government**
1	**PROMO 1**: Restricting the promotion of unhealthy foods: audiovisual media **PROMO 2**: Restricting the promotion of unhealthy foods: non-broadcast media **PROMO 3**: Restricting the promotion of unhealthy foods: Children's environment	Elaborate regulations prohibiting the advertising/promotion of unhealthy foods in children's living environments (schools, sports grounds, playgrounds), in the audiovisual media and in the non-broadcast media.
2	**COMP 3**: Obligatory food enrichment programs	Making it an obligation to comply with food standards, in particular for infant formula and packaged water
3	**PROV 6**: National policy to promote access to WASH	Reinforce the provision of drinking water and sanitation services in line with national standards
4	**PROV 5**: Policies and/or regulations facilitating breastfeeding	Creating space in workplaces to make it easier to look after infants
5	**COMP 3**: Obligatory food enrichment programs	Adopt a monitoring plan for food enrichment programs, including infant flour
6	**PROV 1**: Education policies encourage healthy food choices	Increase the supply of varied and balanced meals based on local produce in school canteens
7	**PROV 1**: Education policies encourage healthy food choices	Reinforce the inclusion of nutrition education in curricula and its promotion in schools
8	**PROV 5:** Policies and/or regulations facilitating breastfeeding	Increase the duration of breastfeeding hours from one and a half hours per day to 3 hours per day for breastfeeding mothers aged between 0 and 6 months.
9	**PROMO 4**: Policies restricting the marketing of Breastmilk Substitutes/broadcast and non-broadcast media	Effective application of current legislation on breast-milk substitutes
10	**TRADE 2**: Protecting regulatory capacity - nutrition	Systematic monitoring (health parameters) of stocks of food of animal origin entering the territory

**Table 3:** Priority infrastructure support actions across all criteria to be recommended to the Burkina Faso government, 2023.

**Order number**	**Indicators concerned**	**Priority actions to be recommended to the government**
1	**LEAD 6**: National breastfeeding Policy	Reinforcing the transition to the scale of infant and young child nutrition
2	**LEAD 2**: Dietary intake targets for the population are defined	Dietary intake targets for the population are defined
3	**LEAD 1**: Strong, visible political support	Constitutionalizing the right to healthy, varied and balanced food
4	**LEAD 9**: Strong and visible political support for undernourishment	Reinforcing actions to fight malnutrition
5	**LEAD 1**: Strong, visible political support	Increase the line's budget allocation for nutrition
6	**LEAD 3**: Implemented food guidelines	Doing a food consumption survey followed by national dietary recommendations
7	**MONIT 2**: Monitoring nutritional status and food intake	Assess the level of implementation of good hygiene practices in school canteens and community restaurants
8	**MONIT 9**: Food safety indicators and standards are defined and monitored	Elaborate a regulatory text on Food Safety
9	**GOVER 3**: Transparency for the public in the development of food policies	Reinforcing the inclusive process in the development of food and nutrition policies
10	**MONIT 7**: Indicators on breastfeeding and complementary breastfeeding monitored	Reinforce actions implemented in connection with complementary food

In the policy component, 10 selected priority actions from the 70 policy actions were identified (Table 2).

In the infrastructure support component, 10 selected priority actions were identified out from the 53 infrastructure support actions (Table 3).

## 4 Discussion

In West Africa, Ghana and Senegal have implemented studies following the results of Food EPI implementation recommendations. In Ghana, for example, two studies were carried out, one on food promotion (commercial food advertising on the campus of Ghana's largest university) and the other on commercial food advertising (food health on the promotional leaflets of fast-food outlets located in Accra's shopping malls). And in Senegal, following on from the Food EPI study, the national consumption survey has been carried out and the data will be released shortly. In addition, a study is currently being carried out on food promotion (exposure of school-age children to unhealthy food advertising in public and media spaces in urban Senegal).

In Burkina Faso, previous analysis of the level of implementation of Food-EPI indicators has identified priority actions targeting gaps in policy or infrastructure implementation. This enabled to evaluate the priorities suggested for the government's future action. Indeed, the experts reached a consensus on the priority actions to be implemented by the government of Burkina Faso, and 123 actions were prioritized, out of them, 10 in policy and 10 in infrastructure support were judged to be the most important, the most feasible and to have a beneficial effect on the double burden of malnutrition, gender and sustainability.

### 4.1 Policy component

Although the prioritization of actions is specific to each country, the priority policy actions above from our study are almost similar to those in Senegal, Ghana and Kenya. Indeed, in these countries, the priority actions selected included raising taxes on unhealthy foods, limiting the promotion of unhealthy foods to children (through broadcast media, non-broadcast media, and rallies), front-of-pack labeling and compositional targets for processed foods, and healthy school food policies (26).

It's also important to note that over forty jurisdictions in more than twenty countries have introduced taxes on sugary drinks, and at least eighteen countries have imposed mandatory restrictions on the advertising of unhealthy foods to children via broadcast or non-broadcast media (11). In view of the impact of food promotion on dietary behavior and consequently on public health, the need to tackle food promotion targeting children must be reinforced (27). Various reports indicate that intensive marketing of fast food and energy-dense, nutrient-poor foods and beverages is a “possible” cause of weight gain and obesity in children (27, 28).

The academic, civil society and public sector experts involved in this study can make a significant contribution to creating a healthy food environment in Burkina Faso by implementing the study's recommendations (11). However, conflicts of interest in the food sector may limit the active participation of some actors in decision-making (29, 30). For this reason, in Burkina Faso, a continuous and extended dialogue between all parties involved, including representatives of the food industry, would be most advisable.

These actions mainly fill major gaps in the country concerning the provision of access to potable water and sanitation, regulations on food promotion and marketing, particularly to children, the introduction of nutrition and food-related subjects in educational establishments, and the importance of providing healthy school meals. In Burkina Faso, current law outlines general provisions, but does not specifically consider the impact of advertising unhealthy foods to children through broadcast or non-broadcast media (31). There is a lack of policies that clearly define which foods are allowed for advertising or in the school environment and which are not. However, these recommended actions will in part enable Burkina Faso to improve the food environment for children, as school food environments have previously been associated with spaces that can offer children the opportunity to develop healthy eating habits that can be transferred to adulthood (32).

In addition, the government should take actions for children under 6 months of age, to enable mothers to benefit from favorable conditions and carry out exclusive breastfeeding until the child is 6 months old, as suggested by the World Health Organization. Several strategies are being deployed in Burkina Faso to promote exclusive breastfeeding and all aspects of regulations on breast-milk substitutes, however, these actions can be reinforced. These include the “stronger with breast milk only” campaign, which aims to mobilize partners, businesses, communities and families to ensure that mothers receive the appropriate information and support they need to adopt exclusive breastfeeding and give their children the best start in life (33). Burkina Faso also has laws and policies governing maternity leave and the protection of pregnant women, based on international labor organization conventions. Indeed, in Burkina Faso two laws include provisions on maternity leave (34) (law 028-2008/AN of May 13, 2008 on labor code and law 081-2015/CNT of November 24, 2015 on the general status of the state civil service).

The priority action to be implemented is to strengthen the provision of access to potable water and sanitation services in line with national standards, because access to potable water, hygiene, and sanitation services remains limited due to the dramatic consequences of the security crisis the country is experiencing. In addition, even if the actors involved are making efforts, the rate of progress toward achieving the objectives, particularly with regard to sanitation, remains low. Added to this is the practice of open defecation, which is still widespread and persistent in communities (35).

The actions to be recommended to the government concerning the composition of foodstuffs specifically include: (i) mandatory compliance with food standards, in particular for infant formula and packaged water, and (ii) the adoption of a monitoring plan for food fortification programs, including infant formula. These actions will highlight the importance the country attaches to compulsory food fortification programs, particularly as regards infant flour, since providing young children with a porridge of good bacteriological and nutritional quality is a means of improving the nutritional status of developing countries (36). In addition, small-scale infant meal production companies are emerging in many developing countries, making it crucial to assess these products from a normative point of view to ensure their survival and positive impact on the quality of infant nutrition.

As far as trade and investment are concerned, the government has been recommended to carry out systematic checks (health parameters) on stocks of animal products entering the country. It is important for the government to adopt measures to manage investments and protect its regulatory capacity in the public health nutrition field.

### 4.2 Infrastructure support component

These actions from the infrastructure support component to be recommended to the Burkinabe government will mainly address gaps in nutrition policy leadership, more specifically in the fight against malnutrition, food guidelines and nutrition financing. Government monitoring and evaluation systems and governance are among the priority domains for which efforts will need to be made in terms of food and nutrition policy development, food safety and good hygiene practices.

Even though Burkina Faso has had a plan for scaling up the promotion of optimal infant and young child feeding practices (2013–2025) since 2013, it has to be said that there are still many efforts to be made, as of April 2021, only 8 of the country's thirteen regions were covered, with varying levels of implementation.

In terms of political leadership, one of the actions focuses on the constitutionalization of the right to a healthy, diversified and balanced diet. According to the WHO, the right to adequate food goes beyond kilocalories. Everyone should have permanent access to healthy, nutritious and culturally acceptable food (37). The SUN (Scaling Up Nutrition) movement, founded on the principle that everyone has the right to food and good nutrition, campaigns for nutrition to be considered a priority on the national political agenda, a cause that has reached many new member countries, such as Burkina Faso in 2011 (38).

According to WHO/UNICEF recommendations, the initiation of early breastfeeding and exclusive breastfeeding are optimal practices whose impact on the survival, growth and development of infants and young children has a significant impact on the overall reduction of neonatal mortality.

In this context, the actions to be recommended to the government concerning the reinforcement of the scaling-up of Infant and Young Child Feeding and the strengthening of actions implemented in the fight against malnutrition will thus enable Burkina Faso, among other things, to achieve its objectives of reducing stunting in children aged under 5 by 2025.

As for the last two actions to be recommended to the government concerning the country's political leadership, these relate to the development of a national food composition table and the carrying out of a food consumption survey followed by national dietary recommendations. These two actions are timely recommendations to the government, as Burkina Faso has not set clear dietary intake targets for its population in terms of nutritional elements of concern, in order to meet WHO recommended dietary intake levels. Also, the non-constant frequency of population surveys (STEPS survey: WHO survey of NCDs risk factors 2013 and The National Iodine and Anemia Survey 2014) was not conducive to an understanding of consumption levels of nutritional elements of concern. With regard to dietary guidelines, WHO recommends that countries implement cross-sectoral, multidisciplinary national policies and action plans aimed at reducing consumption of nutrients of concern (sodium, sugar, and fat), and develop national dietary guidelines 20–22 (39–41). Some African countries, such as Benin, Ghana and Kenya, have their own national dietary guidelines. In Brazil, the Minister of Health has shown leadership by developing new dietary recommendations that are radically different from most of the recommendations developed to date in other countries, and is one of the most widely cited health recommendations. Burkina Faso should also follow the example of some African countries and develop its own national dietary recommendations and guidelines based on food consumption surveys.

A final priority action of political leadership to be recommended to the government concerns a reinforcement of the budget allocation of the nutrition line in Burkina Faso. Promoting the issue of nutrition requires significant and appropriate financial investment, as well as efficient use of available resources.

Donors do not have the resources to meet development challenges on all fronts. Thus, there is an increasingly urgent need to mobilize domestic resources, which means encouraging authorities in developing countries to invest local resources in human capital (i.e., health, nutrition, and education). In Burkina Faso, the government made commitments in 2021 concerning the financing of nutrition at the Nutrition for Growth (N4G) summit, these include (42): increasing spending on nutrition by technical and financial partners from 4% between 2016 and 2018 to at least 10% between 2018 and 2024; zero-rating products used in malnutrition prevention and management inputs by 2024 in 2018 to at least 3% of the national budget by 2024; and increasing the State's contribution to nutrition to 3% (2021).

The three actions relating to monitoring and evaluation systems include a suggestion to the government relate firstly to: evaluate the level of implementation of good hygiene practices in school canteens and community restaurants; carrying out this action will enable the government to ensure that school canteens and community restaurants comply with good hygiene practices. The next step is to draw up a regulatory text on food safety. This action represents a logical follow-up to the political will to reinforce and improve the state of food safety. Indeed, the existence of the national food safety emergency response plan is fully in line with the multi-sectoral national nutrition policy (2020–2029), which gives pride of place to its axis 4 on strengthening food safety. The national food safety emergency response plan is a reference for the competent authority in implementing responses to food safety emergencies (43). The process has already begun at the executive secretariat of the national food safety council, with the support of the African union commission. In conjunction with this is a country food safety profile document, the process of which has already begun at the executive secretariat of the national food safety council, with the support of the African union commission. Finally, the last action concerns the reinforcement of actions implemented in connection with complementary feeding. Increasingly convincing scientific studies show that during the first 1,000 days of life, nutrition, lifestyle habits and other environmental elements have a significant impact on physiology, function, health and future performance. Thus, during the first 2 years of life, it is essential to implement healthy feeding practices for infants to promote healthy growth (44).

With regard to this action in Burkina Faso, there is the Scaling-up plan for the promotion of optimal infant and young child feeding practices (2013–2025), which monitors breastfeeding and complementary feeding indicators (45). Added to this in Burkina Faso there is a communication strategy for social and behavioral change in favor of dietary diversification for children aged 6–23 months. Added to this in Burkina Faso there is a communication strategy for social and behavioral change in favor of dietary diversification for children aged 6–23 months. However, several actions can be implemented, following the example of Asia Pacific, which developed a guideline on complementary nutrition for the Asia-Pacific region during 2020. Kenya and Zambia have also developed complementary food recipe books in their countries (46).

In terms of governance, the action suggested to the government is to strengthen the inclusive process in the development of food and nutrition policies. Indeed, in the policy documents available and accessible in Burkina Faso, none has made it possible to find documented and inclusive actions that value the use of community opinions in the development of food and nutrition policies. However, the multi-sectoral approach is a mechanism which, when implemented, enables the opinions of each informed sector to be gathered more widely during the process of developing certain national food policies. Thus, through decree N°2017/958/MS/CAB concerning the creation, composition, attributions and functioning of Functional Team 5 “multisectoral nutrition management,” the creation of the functional team with the provision of focal points in the various sensitive and specific nutrition sectors makes it possible to have accurate data and information (47).

### 4.3 Limits and strengths

The strength of the approach lies in the use of the Food-EPI tool, which is a rigorous, comprehensive and internationally harmonized methodological framework. It enables an in-depth analysis of the current policy landscape on food environments in the country where it is implemented, and the presence of multi-disciplinary and multi-sectoral stakeholders in the evaluators contributes to government ownership of the results. Added to this is the introduction of two new prioritization criteria to consider gender and sustainability in the actions to be recommended to the government.

The main limitation of the approach used is the sample size, but it may well compare well with similar studies carried out in other countries. Participants were identified according to their skills, and some state actors were replaced by others throughout the process, making the groups less homogeneous than groups of independent actors. Finally, the time devoted to prioritizing actions according to the established criteria was insufficient, because of the number of actions to be prioritized, and also because of the difficulty for the experts to prioritize the importance of the actions, due to the fact that they are equivalent.

## 5 Conclusions

This final step in prioritizing actions to create healthy food environments in Burkina Faso, using the Food-EPI tool, focuses on the efforts needed to improve the safety of the food environment.

In terms of the policy component, it is essential to guarantee access to potable water and sanitation, put in place regulations on food promotion and marketing, particularly for children, introduce nutrition, and food-related subjects into educational establishments, and focus on the provision of healthy food in schools. For the infrastructure support component, priority initiatives focus on political leadership in nutrition, with an accent on the fight against malnutrition, dietary recommendations, and nutrition financing. Governance and governmental monitoring and evaluation systems are essential areas in which efforts will need to be made, particularly with regard to the development of food and nutrition policies, food safety and governance.

For the government to implement these actions, a wide variety of actors, including political decision-makers, civil society, and academia, will be important.

The implementation of Food-EPI in Burkina Faso was useful in developing a consensus for priority action supported by a group of national experts for the creation of healthy food environments in the country. This study represents the latest step in the evaluation of public policies and government actions for the creation of healthy food environments in Burkina Faso using the Food-EPI tool. It highlights required efforts to improve the healthiness of the food environment by identifying and prioritizing actions for healthier food environment in the country.

The activities performed the government are ambitious, and can only be efficient through cooperation between public and non-public actors.

This contribution supports calls for a healthier food environment, and helps strengthen the government's support for decisions to create a healthier food environment.

## Data availability statement

The original contributions presented in the study are included in the article/Supplementary material, further inquiries can be directed to the corresponding author.

## Author contributions

VT: Validation, Writing – original draft, Writing – review & editing, Data curation, Formal analysis, Investigation. CE: Conceptualization, Data curation, Methodology, Project administration, Resources, Supervision, Validation, Writing – original draft. AZ: Conceptualization, Data curation, Methodology, Project administration, Supervision, Validation, Writing – original draft, Resources. JS: Conceptualization, Methodology, Project administration, Resources, Supervision, Validation, Writing – original draft, Data curation. JM: Conceptualization, Funding acquisition, Methodology, Project administration, Supervision, Validation, Writing – original draft. AD: Conceptualization, Funding acquisition, Methodology, Project administration, Supervision, Validation, Writing – original draft. J-CM: Conceptualization, Funding acquisition, Methodology, Project administration, Supervision, Validation, Writing – original draft. SV: Conceptualization, Funding acquisition, Methodology, Project administration, Supervision, Validation, Writing – original draft. MD: Resources, Supervision, Validation, Writing – original draft, Formal analysis.
