# Supplementary material for: Food environment in Burkina Faso: priority actions recommended to the government using Food-EPI tool
Source: Front Nutr. 2024 Jul 18;11:1420323. doi: 10.3389/fnut.2024.1420323 (PMC11293057; doi:10.3389/fnut.2024.1420323)
Supplement: Supplementary file 1 [file Table_1.DOCX]

***Supplementary Material 1***

**Food environment in Burkina Faso: priority actions recommended to the government using Food*‐*EPI tool**

Viviane Aurelie TAPSOBA^*^, Ella COMPAORE, Augustin Nawidimbasba ZEBA, Jerome SOME, Julien Soliba MANGA, Adama DIOUF, Jean- Claude MOUBARAC, Stefanie VANDEVIJVERE and Mamoudou DICKO

*** Correspondance:**  Viviane Aurélie TAPSOBA, E-mail : [viviane.tapsoba@ujkz.bf](mailto:viviane.tapsoba@ujkz.bf)

**Food-EPI tool and adaptation to Burkina Faso**

The good practice indicators contained in these domains cover the policies and infrastructure support needed to improve the healthiness of food environments in order to prevent malnutrition in all its forms and diet-related non-communicable diseases3.

# **The political component**

The Food-EPI tool is made up of a "policy" component with domains that address specific aspects of food environments,

The "food policy" section comprises seven domains that can be implemented to create a healthier food environment. These domains are as detailed below:

**1. Food composition domain**

**Food-EPI vision statement**: There are government systems implemented to ensure that, wherever practicable, processed foods energy and out-of-home meals minimize the energy density and the nutrients of concern (sodium, total fat, saturated fat, trans fat, added sugar).

**This domain has three good practice indicators**: **COMP1**: Targets/standards/restrictions on the composition of processed foods, **COMP2**: Targets/standards/restrictions on the composition of foods eaten outside of the home, and **COMP 3** **(new indicator)**: Fortification programs.

**2. Food labeling domain**

**Food-EPI vision statement**: There is a regulatory system implemented by the government consumer-oriented labeling on food packaging and menu boards in restaurants to enable consumers to easily make informed food choices and prevent misleading claims.

**This domain has four good practice indicators**: **LABEL1**: Ingredient lists / nutrient declarations, **LABEL2**: Regulation of health and nutrition claims, **LABEL3**: Front of pack labeling and **LABEL4**: Menu labeling.

**3. Food promotion domain**

**Food-EPI vision statement/food promotion**: There is a comprehensive policy is implemented by the government to reduce the impact (exposure and power) of unhealthy food promotion to children across all media.

**This domain has three good practice indicators**: **PROMO 1**: Restricting the promotion of unhealthy foods: broadcast media, **PROMO 2**: Restricting the promotion of unhealthy foods: non-broadcast media, **PROMO 3**: Restricting the promotion of unhealthy foods: Children's living environment and **PROMO 4** **(new indicator)**: Policies to restrict the marketing of SLM/broadcast and non-broadcast media.

**4. Food prices domain**

**Food-EPI /Food Price Statement**: Food pricing policies (e.g., taxes and subsidies) are aligned with health outcomes by helping to make the healthy food choices the easier and cheaper choices.

**This domain has four good practice indicators**: **PRICE 1**: Reduce taxes on healthy foods, **PRICE 2**: Increase rates on unhealthy foods, **PRICE 3**: Existing food subsidies favor healthy foods and **PRICE 4**: Food-related income support is targeted at healthy foods.

**5. Food provision domain**

**Food-EPI vision statement**: The government ensures that healthy food service policies implemented in government-funded settings to ensure that food provision encourages healthy food choices, and the government actively encourages and supports private companies to implement similar policies.

**This domain has six (6) good practice indicators**: **PROV1**: Policies in educational structures encourage healthy food choices, **PROV2**: Public policies promote healthy food choices, **PROV3**: Support and training systems (public sector managers), **PROV4**: Support and training systems (private companies), **PROV5** **(new indicator)**: Policies and/or regulations facilitating breastfeeding and **PROV6 (new indicator)**: National policies to promote access to WASH.

**6. Food retail domain**

**Food-EPI vision statement**: The government has the power to implement policies and programs to support the availability of healthy foods and limit the availability of unhealthy foods in communities (outlet density and locations) and in stores (product placement).

**This domain has three (3) good practice indicators**: **RETAIL1**: Robust government policies and zoning laws: unhealthy foods, **RETAIL2**: Robust Government policies and zoning laws: healthy foods and **RETAIL3** **(new indicator)**: Incentive policies and rules/regulations to clean up the informal sector food environment.

**7. Food trade and investment domain**

**Food-EPI vision statement**: The government ensures that trade and investment agreements protect food sovereignty, favour healthy food environments, are linked with domestic health and agricultural policies in ways that are consistent with health objectives, and do not promote unhealthy foods.

**This domain has two (2) good practice indicators**: **TRADE 1**: Impact of trade agreements assessed and **TRADE 2**: Protecting regulatory capacity - nutrition.

# **The infrastructure support**

"Infrastructure support" component with domains that address the strengthening of obesity and NCD prevention systems.

Within the "infrastructure support" component, there are six domains that describe support for the government infrastructure that enables the implementation of successful government policy and action. They are as detailed below :

1. **Leadership domain**

**Food-EPI vision statement/ Leadership**: Political leadership ensures that there is strong support for the vision, planning, communication, implementation and evaluation of policies and actions to create healthy food environments, improve population nutrition and reduce diet-related inequalities.

This domain has nine (9) good practice indicators: **LEAD 1**: Strong and visible political support, **LEAD 2**: Population food intake targets are defined, **LEAD 3**: Food guidelines implemented, **LEAD 4**: Comprehensive implementation plan linked to state/national needs, **LEAD 5**: Priorities for reducing inequalities, **LEAD 6** **(new indicator)**: National breastfeeding policy, **LEAD 7** **(new indicator)**: National complementary feeding policy, **LEAD 8** **(new indicator)**: Exclusive breastfeeding and complementary feeding targets and **LEAD 9** **(new indicator)**: Strong and visible political support for undernutrition.

1. **Governance domain**

**Food-EPI/ vision statement**: Governments have structures in place to ensure transparency and accountability and encourage broad community participation and inclusion when formulating and implementing policies and actions to create healthy food environments, improve population nutrition and reduce diet-related inequalities.

**This domain has four (4) good practice indicators**: **GOVER 1**: Restriction of commercial influence on policy-making, **GOVER 2**: Use of evidence in food policies, **GOVER 3**: Transparency for the public in food policy-making, **GOVER 4**: Access to government information.

1. **Monitoring and evaluation domain**

**Food-EPI vision statement**: The government’s monitoring and intelligence systems (surveillance, evaluation, research and reporting) are comprehensive and regular enough to assess the status of food environments, population nutrition and diet-related NCDs and their inequalities, and to measure progress on achieving the goals of nutrition and health plans.

**This domain has nine (9) good practice indicators**: **MONIT 1**: Monitoring food environments , **MONIT 2**: Monitoring nutritional status and dietary intake, **MONIT 3**: Monitoring body mass index (BMI), **MONIT 4**: Monitoring risk factors and prevalence of NCDs, **MONIT 5**: Evaluation of major programs, **MONIT 6**: Monitoring progress in reducing health inequalities, **MONIT 7** **(new indicator)**: Breastfeeding indicators and complementary monitoring, **MONIT 8** **(new indicator)**: Growth promotion surveillance programs monitored and **MONIT 9** **(new indicator)**: SSA indicators and standards are defined and monitored.

1. **Funding and resources domain**

**Food-EPI vision statement**: Sufficient funding is invested in "Population Nutrition" (estimated from investments in the promotion of healthy eating and healthy food environments for the prevention of obesity and NCDs) to create healthy food environments, improved population nutrition and reductions in obesity, diet-related NCDs and their associated inequalities.

This excludes funding investments in individual promotion (e.g., primary care, prenatal services, maternal and child nursing services, etc.), food security, micronutrient deficiencies (e.g., folate fortification) and undernutrition.

**This domain has three (3) good practice indicators**: **FUND 1**: Population nutrition budget, **FUND 2**: Funding for research into obesity and prevention of NCDs, **FUND 3:** Health promotion agency.

1. **Platforms for interaction domain**

**Food-EPI vision statement**: There are platforms for coordination and opportunities for synergies between departments or ministries, levels of government and other sectors (NGOs, private sector and academia) so that food and nutrition policies and actions are coherent, efficient and effective in improving food environments, population nutrition, diet-related NCDs and their related inequalities.

**This domain has four (04) good practice indicators**: **PLATF1**: Coordination mechanisms (national, state and local government), **PLATF2**: Platforms for interaction between government and the food sector, **PLATF3**: Interaction platforms between government and civil society and **PLATF4**: Integrated and sustainable systems approach with local organizations.

1. **Health-in-all-policies domain**

**Food-EPI vision statement**: Processes are in place to ensure policy coherence and alignment, and that population health impacts are explicitly considered in government policy-making.

**This domain has one good practice indicator**: **HIAP 1**: Assessing the health impacts of food policies.
