# Supplementary material for: Food environment in Burkina Faso: priority actions recommended to the government using Food-EPI tool
Source: Front Nutr. 2024 Jul 18;11:1420323. doi: 10.3389/fnut.2024.1420323 (PMC11293057; doi:10.3389/fnut.2024.1420323)
Supplement: Supplementary file 2 [file Table_2.DOCX]

***Supplementary Material 2***

**Food environment in Burkina Faso: priority actions recommended to the government using Food*‐*EPI tool**

Viviane Aurelie TAPSOBA^*^, Ella COMPAORE, Augustin Nawidimbasba ZEBA, Jerome SOME, Julien Soliba MANGA, Adama DIOUF, Jean- Claude MOUBARAC, Stefanie VANDEVIJVERE and Mamoudou DICKO

*** Correspondence:**  Viviane Aurélie TAPSOBA, E-mail : [viviane.tapsoba@ujkz.bf](mailto:viviane.tapsoba@ujkz.bf)

**Actions identified**

On the basis of the five criteria used in this study, one hundred and twenty-three (123) actions have been defined and presented in the tables below:

# **Actions of the political component**

**Supplementary Table 1**: seventy (70) priority policy actions recommended to the government according to all criteria

| **Order number** | **Policy** | **Indicators** | **Actions to be recommended to the government** | **Average score awarded (%)** |
| --- | --- | --- | --- | --- |
| 1 | PROMO 3.1 | **PROMO 3** | Develop regulations prohibiting the advertising of unhealthy foods in children's living environments (schools, sports fields, playgrounds). | 78,4 |
| 2 | COMP 3.2 | **COMP 3** | Mandatory compliance with food standards, including infant formula flour | 77,4 |
| 3 | PROV 6.4 | **PROV6** | Reinforce the construction of drinking water and sanitation facilities | 76,9 |
| 4 | PROV 5.3 | **PROV5** | Creating spaces in workplaces to facilitate infant care | 76,5 |
| 5 | COMP 3.1 | **COMP 3** | Adopt a monitoring plan for food fortification programs, including infant flour | 75,6 |
| 6 | PROV 1.1 | **PROV 1** | Strengthen the national school food program (including menus based on local produce, nutritional standards and monitoring tools) | 75,3 |
| 7 | PROV 1.2 | **PROV 1** | Accelerate the scaling-up of nutrition education in school curricula | 75,3 |
| 8 | PROV 5.2 | **PROV5** | Increase breastfeeding hours from 1.5 hours a day to 3 hours a day | 74,8 |
| 9 | PROMO 1.1 | **PROMO 1** | Develop a regulatory text prohibiting the promotion of unhealthy foods: audiovisual media | 74,6 |
| 10 | PROMO 2.1 | **PROMO 2** | Develop a regulatory text prohibiting the promotion of unhealthy foods: non-broadcast media | 74,3 |
| 11 | PROMO 4 | **PROMO 4** | Strict application of current legislation on breast-milk substitutes | 73,6 |
| 12 | TRADE 2.3 | **TRADE 2** | Systematic control (sanitary parameters) of stocks of foodstuffs of animal origin entering the territory | 73,5 |
| 13 | PROV 2.4 | **PROV 2** | Encourage research into the processing and preservation of local products | 73,3 |
| 14 | LABEL 1.1 | **LABEL 1** | Draw up a regulatory text requiring compliance with labelling standards for processed foods | 73,2 |
| 15 | PROV 2.2 | **PROV 2** | Promoting the consumption of local products | 73,2 |
| 16 | RETAIL 1.1 | **RETAIL 1** | Reread decree N2016-926/PRES/PM/MATDC/MCU/MJ/MINEFID/MENAPLN on the protection of the school environment, including foods such as fried foods and fast food. | 72,8 |
| 17 | COMP 2.2 | **COMP 2** | Develop and promote catering standards | 72,4 |
| 18 | PROMO 3.2 | **PROMO 3** | Take stock of the problem of unhealthy foods commercially promoted to children | 72,4 |
| 19 | RETAIL 1.2 | **RETAIL 1** | Train and involve parent-teacher associations and school management committees. | 72,3 |
| 20 | COMP 1 | **COMP 1** | Develop legislation that takes into account the limiting aspects of certain nutrients of concern in processed foods | 71,50 |
| 21 | COMP 2.1 | **COMP 2** | Draw up and disseminate a national directive on collective catering | 71,4 |
| 22 | LABEL 2.1 | **LABEL 2** | Compulsory certificate of conformity for composition and labelling claims | 71,1 |
| 23 | COMP 3.3 | **COMP 3** | Raising awareness of best practices for storing and using fortified foods | 70,9 |
| 24 | PROV 3.2 | **PROV 3** | Strengthen the capacity of restaurateurs/providers in public schools and establishments | 70,4 |
| 25 | PROV 6.3 | **PROV 6** | Adapting toilets to gender | 70,4 |
| 26 | TRADE 2.4 | **TRADE 2** | Strengthen the capacities of technical structures in the diligent control of foodstuffs of animal origin | 70,4 |
| 27 | TRADE 2.5 | **TRADE 2** | Inspecter régulièrement les stocks de denrées d’origine animale | 70,1 |
| 28 | RETAIL 3.3 | **RETAIL 3** | Regular inspection of stocks of animal products | 69,9 |
| 29 | PRIX 2 | **PRIX 2** | Impose an additional 20% tax on all products deemed unhealthy (sugary drinks, foods high in salt, fat, etc.) | 69,6 |
| 30 | PROV 3.3 | **PROV 3** | Set up a control and monitoring system for public school restaurants | 69,6 |
| 31 | PROV 6.2 | **PROV6** | Create and maintain public toilets | 69,4 |
| 32 | RETAIL 2.2 | **RETAIL 2** | Improving distribution channels for fruit and vegetables from production areas | 69,2 |
| 33 | PRIX 3 | **PRIX 3** | Strengthen subsidies for agricultural producers, especially fruit and vegetable growers, who promote healthy foods | 69 |
| 34 | PRIX 4.3 | **PRIX 4** | Strengthen monitoring of the implementation of the response and support plan for vulnerable populations: food and nutritional assistance component | 69 |
| 35 | LABEL 3.2 | **LABEL 3** | Reread standard NBF 01-117 version 2009, taking into account the presence of the nutritional composition of foods on the label of packaged foods. | 68,8 |
| 36 | PROV 2.1 | **PROV 2** | Evaluate and review the implementation of Order N2017-002/PM/CAB of January 31, 2017 on the purchase of local food products by state structures as part of their procurement. | 68,8 |
| 37 | PRIX 4.2 | **PRIX 4** | Increase the national budget allocated to food assistance targeting healthy foods | 68,7 |
| 38 | TRADE 2.2 | **TRADE 2** | Respecting international agreements on nutrition; | 68,5 |
| 39 | PROMO 2.2 | **PROMO 2** | Mandate a control structure for food promotion: non-broadcast media | 68,4 |
| 40 | PROV 2.6 | **PROV 2** | Analyze the salt and sugar content of menus served in hospitals and school canteens | 68,3 |
| 41 | PROMO 1.2 | **PROMO 1** | Mandate a structure to monitor food promotion: audiovisual media | 67,9 |
| 42 | PRIX 1.2 | **PRIX 1** | Reinforce price controls on healthy foods | 67,6 |
| 43 | PROV 2.3 | **PROV 2** | Recruit nutritionists and food technologists for the decentralized structures of Burkina Faso's Ministry of Education, Literacy and Promotion of National Languages. | 67,6 |
| 44 | COMP 3.4 | **COMP 3** | Reinforce pre- and post-marketing control of fortified foods | 67 |
| 45 | RETAIL 3.1 | **RETAIL 3** | Mapping healthy food outlets at community level | 67 |
| 46 | RETAIL 3.4 | **RETAIL 3** | Distinguish between players who apply good practices to all communities | 67 |
| 47 | RETAIL 2.1 | **RETAIL 2** | Extend coverage of fruit and vegetable markets in secondary towns throughout the country | 66,6 |
| 48 | PRIX 1.1 | **PRIX 1** | Set up a system to reduce taxes on imported healthy foods not available locally | 66,4 |
| 49 | RETAIL 3.5 | **RETAIL 3** | Penalizing offenders | 66,4 |
| 50 | PROV 5.1 | **PROV 5** | Increase the number of months of maternity leave from 3 to 6 months | 66,2 |
| 51 | PROV 6.1 | **PROV 6** | Scale up community-led Total Sanitation strategy (End of Open Defecation) | 66,2 |
| 52 | LABEL 4.2 | **LABEL 4** | Raising awareness of the regulatory framework for menu labeling | 66,1 |
| 53 | LABEL 2.2 | **LABEL 2** | Impose criminal sanctions on perpetrators of unfounded allegations | 66 |
| 54 | PRIX 4.1 | **PRIX 4** | Set up a subsidy mechanism for healthy foods in food assistance programs | 65,8 |
| 55 | LABEL 1.3 | **LABEL 1** | Check nutrient declarations on labels | 65,7 |
| 56 | COMP 2.3 | **COMP 2** | Identify and issue an accreditation/certification/approval | 65,4 |
| 57 | TRADE 2.1 | **TRADE 2** | Strengthening existing regulatory structures | 65,4 |
| 58 | PROV 3.4 | **PROV 3** | Creating curricula for foodservice | 64,9 |
| 59 | PROV 4.1 | **PROV 4** | Training for restaurateurs/service providers in private establishments | 64,9 |
| 60 | PROV 4.3 | **PROV 4** | Promoting nutrition education in private companies | 64,7 |
| 61 | PROV 2.5 | **PROV 2** | Monitor implementation of the action plan for the national strategy to promote local products | 64,4 |
| 62 | LABEL 3.1 | **LABEL 3** | Develop a label coding system adapted to the Burkina Faso context | 64,3 |
| 63 | RETAIL 2.3 | **RETAIL 2** | Define and locate healthy food outlets at community level | 63,9 |
| 64 | LABEL 1.2 | **LABEL 1** | Reinforce application (training/awareness-raising) of the labelling standard | 63,7 |
| 65 | RETAIL 3.2 | **RETAIL 3** | Develop visual control tools to promote a healthy food environment in the informal sector | 63,6 |
| 66 | TRADE 1.1 | **TRADE 1** | Make the technical departments responsible for impact assessments of trade agreements operational | 63,4 |
| 67 | LABEL 4.1 | **LABEL 4** | Develop regulations on menu labeling for fast-food outlets | 62,6 |
| 68 | PROV 4.2 | **PROV 4** | Reduce the tax burden on private companies promoting healthy foods | 62,4 |
| 69 | TRADE 1.2 | **TRADE 1** | Assessing the impact of trade agreements on the nutritional environment | 60,8 |
| 70 | PROV 3.1 | **PROV 3** | Operating restaurants in structures other than schools and establishments | 59,2 |

# **Infrastructure support actions**

**Supplementary Table 2**: Fifty-three (53) priority policy actions to recommend to the government according to all criteria

| **Order number** | **Policy** | **Indicators** | **Actions to be recommended to the government** | **Average score awarded (%)** |
| --- | --- | --- | --- | --- |
| 1 | LEAD 6.2 | **LEAD 6** | Accelerating ANJE's transition to scale | 76,80 |
| 2 | LEAD 2.2 | **LEAD 2** | Develop a national food composition table | 75,80 |
| 3 | LEAD 6.1 | **LEAD 6** | Implement a specific policy for exclusive breastfeeding | 75,20 |
| 4 | LEAD 1.2 | **LEAD 1** | Constitutionalizing the right to healthy, varied and balanced food | 74,30 |
| 5 | LEAD 9 | **LEAD 9** | Reinforce actions to combat malnutrition | 73,90 |
| 6 | LEAD 1.3 | **LEAD 1** | Strengthen advocacy of a budget line for nutrition | 73,60 |
| 7 | LEAD 3 | **LEAD 3** | Develop national dietary recommendations and guidelines based on food consumption surveys | 73,50 |
| 8 | MONIT 2.1 | **MONIT 2** | Continuously assess the quality of food served in canteens and restaurants | 72,70 |
| 9 | MONIT 9.2 | **MONIT 9** | Drawing up a regulatory text on food safety | 72,40 |
| 10 | GOVER 3.1 | **GOVER 3** | Strengthen the inclusive process in food and nutrition policy-making | 72,10 |
| 11 | LEAD 7 | **LEAD 7** | Establish a specific national policy for complementary foods | 72,00 |
| 12 | MONIT 7 | **MONIT 7** | Reinforce monitoring of breastfeeding indicators and complementary feeding | 71,90 |
| 13 | MONIT 2.2 | **MONIT 2** | Periodically assess the nutritional status of schoolchildren, adolescents and the elderly | 71,80 |
| 14 | LEAD 2.1 | **LEAD 2** | Conduct periodic (5-year) national food consumption surveys | 71,70 |
| 15 | MONIT 9.1 | **MONIT 9** | Tighter controls on pesticides | 71,30 |
| 16 | LEAD 1.1 | **LEAD 1** | Operationalizing institutional anchoring | 71,2 |
| 17 | HIAP 1 | **HIAP 1** | Set up a national mechanism to assess the impact of food policies on health | 71,20 |
| 18 | GOVER 2.2 | **GOVER 2** | Involve learned societies in drafting food policies | 71,00 |
| 19 | GOVER 4.4 | **GOVER 4** | Popularize the information platform on nutrition in Burkina Faso | 70,70 |
| 20 | FUND 1.2 | **FUND 1** | Respect national and international commitments to nutrition | 70,40 |
| 21 | MONIT 3.2 | **MONIT 3** | Accelerate development of the strategic plan for adolescent and youth health | 70,30 |
| 22 | MONIT 3.1 | **MONIT 3** | Accelerate the adoption and implementation of the integrated strategic plan for the fight against non-communicable diseases (2021-2024) | 69,90 |
| 23 | LEAD 5.1 | **LEAD 5** | Strengthening gender mainstreaming | 69,80 |
| 24 | FUND 2.3 | **FUND 2** | Present Nutrition research priorities to the High Council for Research | 69,80 |
| 25 | LEAD 5.2 | **LEAD 5** | Advocate for state funding of the response and support plan for vulnerable populations | 69,60 |
| 26 | MONIT 4.1 | **MONIT 4** | Regular STEPS surveys (5 years) | 69,30 |
| 27 | PLATF 1 | **PLATF 1** | Strengthen the various coordination mechanisms (ST Nut, CNaN, ONEHEALTH platform, etc.) | 69,20 |
| 28 | MONIT 9.3 | **MONIT 9** | Extend standards to all local products | 68,50 |
| 29 | GOVER 4.1 | **GOVER 4** | Disaggregating nutrition data and making it accessible | 68,30 |
| 30 | PLATF 2 | **PLATF 2** | Create a platform for interaction between the government and the food sector (JAAL, FIAB, supermarket association, food retailers, etc.). | 68,30 |
| 31 | LEAD 4 | **LEAD 4** | Develop a comprehensive national implementation plan for improving food environments | 68,00 |
| 32 | GOVER 3.2 | **GOVER 3** | Making information accessible and taking account of beneficiaries' opinions | 68,00 |
| 33 | LEAD 8 | **LEAD 8** | Strengthening the implementation of existing programs | 67,90 |
| 34 | FUND 2.4 | **FUND 2** | Implementing the Research Funding Act | 67,80 |
| 35 | GOVER 2.1 | **GOVER 2** | Making data accessible across institutions | 67,70 |
| 36 | FUND 3 | **FUND 3** | Provide health promotion structures with the necessary material and financial resources | 67,40 |
| 37 | MONIT 1 | **MONIT 1** | Extend food environment monitoring to all public sectors | 67,30 |
| 38 | FUND 2.2 | **FUND 2** | Advocate the alignment of the national research and innovation fund to research with nutrition programs in sectoral research programs | 67,20 |
| 39 | MONIT 8 | **MONIT 8** | Strengthening growth monitoring programs | 67,10 |
| 40 | FUND 2.1 | **FUND 2** | Dedicate funds from the national research and innovation fund to research into obesity and the prevention of NCDs. | 66,70 |
| 41 | PLATF 4.2 | **PLATF 4** | Introduce a local food environment day | 66,70 |
| 42 | GOVER 4.3 | **GOVER 4** | Regularly update the websites of government departments and services | 66,30 |
| 43 | MONIT 4.2 | **MONIT 4** | Expand the panel of NCDs in the STEPS survey to include cancers, chronic kidney disease, etc. | 66,20 |
| 44 | MONIT 6.2 | **MONIT 6** | Strengthen the implementation of projects and programs aimed at reducing health inequalities | 65,60 |
| 45 | PLATF 3 | **PLATF 3** | Set up a platform for interaction between government and civil society | 65,60 |
| 46 | GOVER 4.2 | **GOVER 4** | Popularize data from national-language documents | 65,00 |
| 47 | MONIT 5 | **MONIT 5** | Setting up a national assessment mechanism | 64,40 |
| 48 | GOVER 1.1 | **GOVER 1** | Put in place a robust regulatory framework to restrict commercial influence on policy-making related to food environments | 63,00 |
| 49 | MONIT 6.1 | **MONIT 6** | Strengthen systems for monitoring progress in reducing health inequalities | 62,30 |
| 50 | FUND 1.1 | **FUND 1** | Specify budget lines | 61,00 |
| 51 | PLATF 4.1 | **PLATF 4** | Strengthen the effective transfer of resources to communes | 59,10 |
| 52 | GOVER 1.3 | **GOVER 1** | Drawing up a reference document on conflicts of interest | 57,30 |
| 53 | GOVER 1.2 | **GOVER 1** | Set up a mandatory, publicly accessible lobbying register | 54,60 |
